# The GapMap project: a mobile surveillance system to map diagnosed autism cases and gaps in autism services globally

**DOI:** 10.1186/s13229-017-0163-7

**Published:** 2017-10-23

**Authors:** Jena Daniels, Jessey Schwartz, Nikhila Albert, Michael Du, Dennis P. Wall

**Affiliations:** 1 Department of Pediatrics, Division of Systems Medicine, Stanford University, Stanford, CA USA; 2 Department of Biomedical Data Science, Stanford University, Stanford, CA USA; 3 Department of Computer Science, Princeton University, Princeton, NJ USA; 4 1265 Welch Rd X141, Stanford, CA 94305 USA

**Keywords:** Autism, Autism spectrum disorder, Crowdsourcing, Prevalence, Resources, Epidemiology

## Abstract

Although the number of autism diagnoses is on the rise, we have no evidence-based tracking of size and severity of gaps in access to autism-related resources, nor do we have methods to geographically triangulate the locations of the widest gaps in either the US or elsewhere across the globe. To combat these related issues of (1) mapping diagnosed cases of autism and (2) quantifying gaps in access to key intervention services, we have constructed a crowd-based mobile platform called “GapMap” (http://gapmap.stanford.edu) for real-time tracking of autism prevalence and autism-related resources that can be accessed from any mobile device with cellular or wireless connectivity. Now in beta, our aim is for this Android/iOS compatible mobile tool to simultaneously crowd-enroll the massive and growing community of families with autism to capture geographic, diagnostic, and resource usage information while automatically computing prevalence at granular geographical scales to yield a more complete and dynamic understanding of autism resource epidemiology.

## Background

Across the globe, families affected by autism must navigate substantial gaps in availability of health care resources. The incidence of autism has been on the rise in the USA and across the globe, but the rate and exact prevalence of autism remain unclear. Estimates from the Centers for Disease Control (CDC) represent extrapolations from a small collection of states, and by the time of publication, they can be up to 2 years out of date. Today’s alarming estimate of 1 in 68 children is based on data from 2014 [1, 2]. Actual rates are likely to be even higher, but until we have fully federated electronic medical records that are accessible on a national scale, we will not have a robust understanding of the true prevalence in the US let alone on a global scale for this life-long condition.

As the number of autism diagnoses has increased, so too has the number of children with a high risk for autism that remain undiagnosed [3]. This translates into a waiting period estimated to average 13 months for a diagnostic assessment in the USA [4]. These wait times are even longer in rural environments and underserved areas with lower socioeconomic status [3–7]. Disparities in geographic coverage of resources often coincide with a lack of public awareness of autism spectrum disorders (ASD) and difficulty in finding diagnostic or post-diagnosis options with appropriate insurance coverage, exacerbating diagnostic delays and increasing the amount of hassle and stress for families navigating complexities associated with an ASD diagnosis throughout that individual’s life. The resulting delays in diagnoses are also detrimental to children who are unable to receive behavioral and intervention therapies during the critical periods in which they are maximally impactful [8–12]. Frustratingly, however, we do not yet know the exact size and severity of gaps in access, where the widest divides exist globally, and the specific types of disparities across the life course as individuals with autism transition to adulthood.

## Main text

Little attention has been paid to creating curated, easily Web-searchable, and comprehensive lists of autism services. The few exceptions include Autism Speaks [13] and Autism Source [14], yet these account for only about 1500 unique resources, likely a fraction of the actual.

### Resource gaps

Resource gaps (regions in which there exist limited diagnostic or treatment resources with respect to the demand) require comprehensive knowledge of both autism epidemiology and the geographic distribution of autism resources. Finding and understanding these resource gaps can drive novel innovation of products that can mobilize to the home and/or create shifts in resource usage to direct jobs and care towards particularly fillable gaps in care management for individuals with autism. This can be done through collecting robust hard data, allocating resources more efficiently, and providing information to emerging organizations and businesses to let them know where their services are needed most.

While autism epidemiology is a common research area [15] and Rzhetsky et al. found that the incidence of ASD is affected by the state-level regulatory and environmental factors [16], we are still far from understanding the true prevalence of diagnosed cases of autism. Many current epidemiological studies suffer from small sample sizes and regional focuses [15, 17]. For example, the CDC determined that the autism prevalence rate in the USA is about 1 in 68 children based on only 11 communities [1]. Additionally, most autism prevalence studies do not include undiagnosed individuals [1, 2, 15, 17]. This means that individuals without access to diagnostic centers for socioeconomic or geographic reasons are not reported, resulting in underrepresented statistics. Since location at the city level is considered personally identifiable information, researchers are generally unable to share data with locations attached, which ultimately precludes greater autism epidemiological understanding and accuracy.

### Geographic disconnect

Understanding the geographic distribution of autism resources is as difficult as understanding resource gaps. Although many ASD resource directories exist, most are for very small regions (at the city or state level) and can have data integrity drawbacks, including a lack of updated information, incomplete information, and missing resources. More importantly, very few ASD resource directories include critical information pertaining to diagnostic capability. The National Autistic Society United Kingdom’s Autism Services Directory [18] is an example of an online resource directory that is autism-specific, relatively comprehensive, and includes key diagnostic information. Replicating such a registry in the USA would not only help complete our understanding of resource distribution, but it would also enable families and individuals with autism to quickly find the best resources near them.

Despite the difficulty, it is still worth finding closer approximations of the geographic distribution of autism and autism resources. Analyses conducted with 47,622 individuals with autism, based on information gathered from online public profiles and social media accounts, and 840 developmental medical centers in the USA, collected through Autism Speaks [13] and Autism Source [14], suggest that resource discrepancies may be much worse than initially thought and that the paucity of resources in various economic communities likely contributes to inequities in a family’s ability to access appropriate and necessary therapies, services, and support [19]. The average distance from an individual with a diagnosis of autism to a diagnostic center was estimated at 32 km, and an astonishing 70% of individuals lived no closer than 30 km of a diagnostic center. Assuming geographic variations in autism prevalence rates are relatively modest, it is possible that a majority of individuals with risk for an autism diagnosis live prohibitively far from a diagnostic center––especially with the uneven allocation of 840 diagnostic centers for a nation of 9.85 million squared kilometers [20]. Most likely, there is a large disconnect between resources and individuals with autism that need an official diagnosis and healthcare services.

### Mobile solution

To complement the resource lists from Autism Speaks and Autism Source, we have devised a tool, GapMap (http://gapmap.stanford.edu), to obtain more accurate and widespread estimates of geographic variations in autism prevalence rates and resource availability. GapMap is a mobile-first website or an application that renders well and is fully usable from a mobile or tablet device but can also be accessed through a traditional computer. Minorities, households with an income of less than $50,000, and the non-college educated are more likely to use mobile Internet as their primary or only device for Internet access [21]. Individuals in rural areas are less likely to access the Internet, with or without cell phones; however, usage is high enough to warrant developing health-related Internet and mobile applications [22–24]. As such, data collected through GapMap will still be able to reduce bias in prevalence data.

**Fig. 1:**
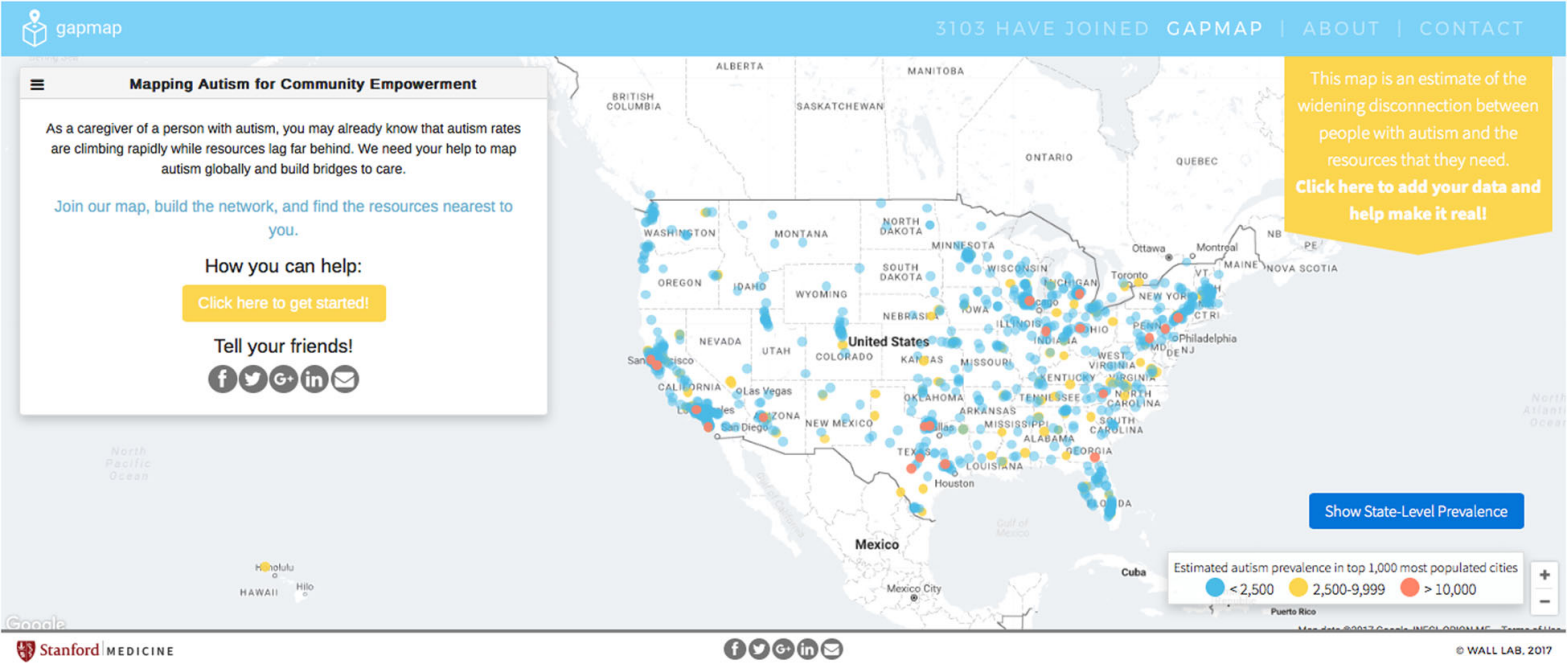
Example of the mapping interface and home page for GapMap

GapMap features a map with overlays of real-time autism prevalence and resource markers. Dynamic features allow visitors to electronically consent, contribute data, find local resources, and learn more about the study. Current estimates of autism prevalence rates have been used to simulate data for the map. Similarly, GapMap’s resource bank already contains extracted data from both regional and national pre-existing online resource directories (including Autism Speaks and Autism Source) [13, 14]. This dataset has been further refined by algorithmic categorization, classification (as a center, specialist, or online resource), and deduplication. See Fig. 1 for GapMap’s interface.

Neither the prevalence data nor the resource banks are complete, but a simple form lets individuals with autism (or caregivers of a child with autism) submit data. These data include gender, date of birth, location (city and state), specific diagnosis/co-morbid conditions, contact information, and local services that have been used. IP addresses, date and time of submission, and similarity of data submitted will be used to detect duplicate or flag anomalies as potentially falsified data. Participants also provide answers to a machine learning behavioral classification system, which has been shown to match clinical diagnostic outcomes with high frequency [25–29]. Crowdsourced data has been shown to match the quality of expert-curated data with proper instructions for data submission and reasonable validation on input data [30–32].

Local services include medical specialists, therapists, support resources, and “autism-friendly” generic services. After submission, locational data will be anonymously incorporated into the prevalence map; all other data will be securely managed and used to better understand autism resource deficits. In the future, site visitors will also be able to easily add to or edit the autism resource bank and fill in ASD-specific information such as diagnostic capability, target age, and accommodated disorders/disabilities. While resource directories are often difficult and costly to maintain, as new services open and others shut down, crowdsourcing offers a lower cost solution: leverage the collective knowledge of individuals providing, using, and seeking resources. In particular, parents of young children are more likely to search for and share information online [33], and resource providers have an incentive to list themselves for discoverability. IP addresses, submitter account information, and contribution activity will be tracked and used to detect malicious users, unusual resource deletions or additions, or questionable resource review submissions. Although there may be an incentive for providers to supply “fake reviews” of their services or their competitors, it is a common crowdsourcing problem with existing spam detection and filtering algorithms [34]. This filtering will ensure that questionable resources are removed. In addition, we will validate any organic resources through a machine learning algorithm that will confirm the contact data that was provided by our users that corresponds to what is publicly available on the Internet. If we do not find a resource-match, we will not include the resource in GapMap’s database. Our hope is that empowering families and individuals to contribute data allows for a robust and constantly updated global database of autism resources and prevalence rates.

### System architecture

**Fig. 2:**
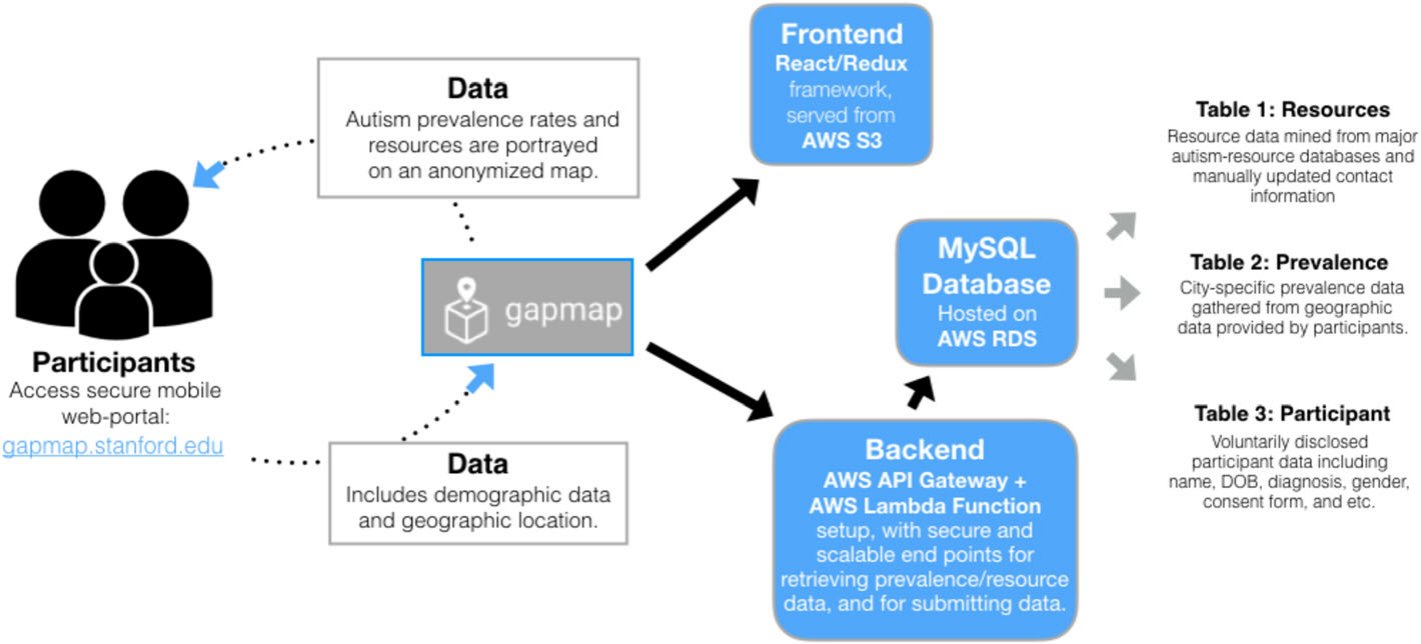
The technical architecture planned for GapMap. The server setup will be fully encrypted and HIPAA compliant to maintain subject data securely on an ongoing basis

Data are encrypted and stored on secure MySQL databases behind a firewall. GapMap is written in React.js and runs on Amazon Web Services Simple Storage Serve (AWS S3). The backend server runs on AWS application program interface (API) Gateway and AWS Lambda. AWS API Gateway executes specific JavaScript packages, novel code that interacts with our SQL database, on AWS Lambda. The MySQL relational database is hosted on Amazon Relational Database Service (RDS) and consists of two main tables. Table 1 holds the resource data, including name/type of resource, geographic coordinates, address, and contact information. Table 2 holds participant data to include specific diagnosis, consent form, geographic location (zip code), and other personally identifiable information. See Fig. 2 for an overview of the planned system architecture.

## Conclusions

If successful, GapMap could help the families of 3.5 million individuals in the USA [35] quickly and stresslessly find services that range from diagnostic evaluations to a variety of forms of therapy. It could help thousands of individuals recently diagnosed with ASD and their families each year find options for therapy, schooling, insurance, and support. It can also help the approximately 27% of children who remain undiagnosed with ASD by age 8 [36] find the best resource options as they progress through school (e.g., from speech therapy to behavioral therapy to cognitive therapy) and also aid 52 million individuals with ASD worldwide [15] find employment, relationships, and independent living support. In addition, GapMap would allow policy experts to advocate in favor of efforts to increase the number of autism-related clinical practices in resource-poor areas and provide large-scale data for researchers studying the causes of and potential therapies for ASD.

### Limitations

There is potential for bias in reporting and data capture that could skew the data incorporated into GapMap over time. To safeguard against biased reporting of autism diagnoses, we have incorporated a set of behavioral questions validated for autism detection by a series of machine learning experiments [26, 28]. We also provide the option to upload a video of the child with autism to enable secondary evaluation of the autism diagnosis using a separate machine learning approach [25, 27, 29]. These approaches maintain a balance between reporting complexity and information content to help ensure high participation while retaining accuracy. To safeguard against incorporation of inaccurate resource information, we will continue to enhance the GapMap software system to (a) automatically check for the functionality of URLs, emails, and phone numbers, and (b) incorporate and prioritize resources that have well-established reputations. For the latter category, we will use Search Engine Optimization (SEO) rankings to flag Web accessible resource listings that have higher numbers of independent links on external sites and prioritize sites associated with highly regarded academic medical centers, as well as sites with clinical researchers who have PubMed indexed and salient publications in the field of autism care. In addition, as the GapMap population grows, we will enable participants to rate sites listed within their geographic area, providing a “Yelp-like” means to crowdsource the process of vetting listed resources. Over time, this will not only include star ratings but also more detailed information like the average reported waiting time to receive services.

Despite these limitations, utilizing crowdsourced data for the prevalence of diagnosed cases of autism and autism-related resources can impact positively on the quality of and access to robust information, through the involvement of an active and increasingly large population [37–39]. GapMap will be an invaluable tool to members of the autism community, as it can inexpensively and feasibly amass valuable information. With this tool and others like it, we will be able to quantify the geographic disconnect that exists worldwide and leverage this information to innovate targeted strategies that give families answers and ability to act faster and with greater frequency.

## Abbreviations

API: AWS application program interface
ASD: Autism spectrum disorders
AWS S3: Amazon Web Services Simple Storage Serve
CDC: The Centers for Disease Control and Prevention
RDS: Amazon Relational Database Service
SEO: Search Engine Optimization
